# Bioinformatics on a national scale: an example from Switzerland

**DOI:** 10.1093/bib/bbx073

**Published:** 2017-07-04

**Authors:** Vivienne Baillie Gerritsen, Patricia M Palagi, Christine Durinx

**Affiliations:** 1 SIB Swiss Institute of Bioinformatics, Communications, 1 Rue Michel Servet, Geneva, Switzerland; 2 SIB Swiss Institute of Bioinformatics, Geneva, Switzerland; 3 SIB Swiss Institute of Bioinformatics, Bâtiment Génopode, Bâtiment Génopode, Lausanne, Vaud, Switzerland

**Keywords:** research and service groups, core facilities, competence centres, clinical bioinformatics, personalized health, infrastructure, training, long-term sustainability

## Abstract

Switzerland has been a pioneer in the field of bioinformatics since the early 1980s. As time passed, the need for one entity to gather and represent bioinformatics on a national scale was felt and, in 1998, the SIB Swiss Institute of Bioinformatics was created. Hence, 2018 marks the Institute’s 20th anniversary. Today, the Institute federates 65 research and service groups across the country—whose activity domains range from genomics, proteomics, medicine and health to structural biology, systems biology, phylogeny and evolution—and a group whose sole task is dedicated to training. The Institute hosts 12 competence centres that provide bioinformatics and biocuration expertise to life scientists across the country. SIB sensed early on that the wealth of data produced by modern technologies in medicine and the growing self-awareness of patients was about to revolutionize the way medical data are considered. In 2012, it created a Clinical Bioinformatics group to address the issue of personalized health, thus working towards a more global approach to patient management, and more targeted and effective therapies. In this respect, SIB has a major role in the Swiss Personalized Health Network to make patient-related data available to research throughout the country. The uniqueness of the Institute’s governance structure has also inspired the structure of other European life science organizations, notably ELIXIR.

## SIB Swiss Institute of Bioinformatics: a brief presentation

Since the early 1980s, Switzerland has been a pioneer in the field of bioinformatics and was swift to recognize both the advantage and the necessity to create an entity, which would gather and represent bioinformatics on the national scale. In the wake of a funding conundrum, five scientists who had been developing bioinformatics resources and software for over a decade decided to join forces and founded the SIB Swiss Institute of Bioinformatics, or SIB (www.sib.swiss) in 1998. Hence, 2018 marks its 20th anniversary.

Today, SIB federates research and service groups as well as competence centres that develop, maintain and provide bioinformatics resources for all areas of the life sciences on the national and the international level. These groups are located throughout Switzerland and are also part of the country’s major schools of higher education and research institutes.

Besides SIB’s core facilities and competence centres, the Institute also supports ‘embedded’ bioinformaticians in research and clinical laboratories—thus offering constant support to biomedical research and data analysis and a sensing mechanism to identify necessary innovation. The Institute offers training courses in many domains of bioinformatics both for researchers in the life sciences and bioinformaticians, and the PhD Training Network has been established.

Acknowledging the growing importance of integrative approaches to personalized health, SIB has already laid down the basis required to collaborate effectively with the country’s clinicians by putting at their disposal the Institute’s bioinformatics and curation expertise. Such a tight collaboration will assist in the comprehensive assessment of diagnosis, and help select the best treatment possible for a given patient. SIB also has a leading and coordinating role in the Swiss Personalized Health Network (SPHN, sphn.ch), which is establishing a national infrastructure to allow the sharing of patient data for research across Switzerland. Within the SPHN, SIB is running the Data Coordination Centre, which supports data interoperability and organization on a national scale.

The SPHN initiative aims to bring Switzerland at the forefront of personalized health research by establishing interoperability of health-related data across Switzerland for research purposes. The infrastructure is to integrate the university hospitals, schools of higher education, research institutes and organizations working in the area of personalized health. Standardization of data semantics, data exchange formats and harmonization of data generation approaches will form the basis for efficient data sharing to enable research projects in personalized health and precision medicine.

## SIB: beginnings, evolution, where it is now

### Beginnings

In 1996, despite its worldwide renown and utility within the life science community, the Swiss-Prot protein sequence knowledgebase came to a near standstill. Funding through research grants was not possible anymore because the knowledgebase was more a service than it was basic research. The Swiss-Prot group was given 2 months to find a solution, without which its database would be forced to come to an end, and all those developing and maintaining it would lose their jobs.

During the weeks that followed >2500 messages of support were received from life scientists all over the world, including several Nobel prize laureates. The Swiss press as well as the journals *Nature* and *Science* relayed the news and, thanks to the backing of a few visionary Swiss politicians, the Swiss-Prot database survived an extra 2 years with interim funding. However, a viable solution needed to be found. The SIB was finally founded on 30 March 1998 by five PIs already active in the field of bioinformatics, and who had been collaborating for many years: Ron Appel then director of the Laboratory for Molecular Imaging and Bioinformatics at the Geneva University Hospitals (ExPASy; SWISS-2DPAGE), Amos Bairoch then head of the bioinformatics group in the Department of Medical Biochemistry at the University of Geneva (SWISS-PROT; PROSITE; ENZYME), Philipp Bucher then head of the bioinformatics group at the Swiss Institute for Experimental Cancer (Eukaryotic Promoter Database, EPD), Victor Jongeneel then director of the Information Technology Unit at the Ludwig Institute (EMBnet) and finally Manuel Peitsch then world leader of Scientific Computing for Glaxo-Wellcome (SWISS-MODEL; SWISS-3DIMAGE). The Swiss-Prot protein sequence knowledgebase was saved and Switzerland had its institute of bioinformatics, i.e. an organization that could federate research and service groups as well as training throughout Switzerland, thus creating a pool of Swiss bioinformaticians whose infrastructure and resources would be open to all [1].

### The way things evolved

**Figure 1:**
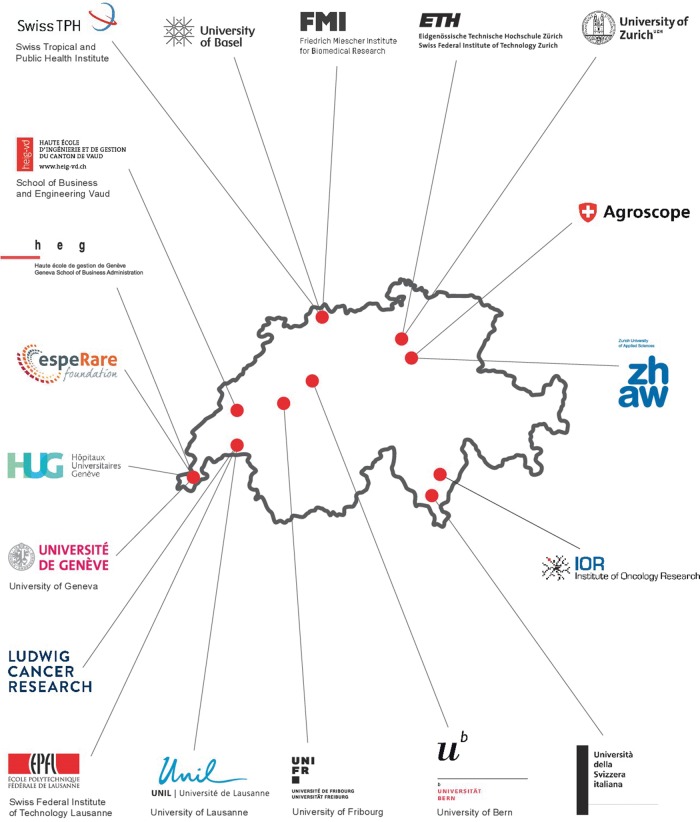
SIB across Switzerland. SIB consists of bioinformatics research and service groups affiliated to the major Swiss schools of higher education and research institutions. Early 2017, SIB counted 65 research groups, service groups and competence centres, and 19 national partner institutions.

Things built up—slowly, but surely. It was becoming obvious that there could be no research in the life sciences without bioinformatics. SIB was already in a leading position in this respect. Little by little, the Institute spread its wings across the country, and the number of members and partner institutions gradually increased. In 20 years, SIB has consolidated its position as a leader in bioinformatics on the national scale, while also paving its way to international recognition. In the first quarter of 2017, SIB counted 65 research groups, service groups and competence centres; about 800 members; 19 national partner institutions; >150 resources—many of them used worldwide—and 12 core facilities. In two decades, the Institute has become the most important federation and national network of academic research and services in the field of bioinformatics in Switzerland (Figure 1).

SIB’s imprint also began to flourish internationally. In particular, in 2007, Switzerland became involved in the construction and implementation of the European Life Sciences Infrastructure for Biological Information, known as ELIXIR, the pan-European initiative to build a sustainable infrastructure for managing and safeguarding biological information throughout Europe. The model of collaboration between the country’s cantons and institutions built up over the years by SIB became the model of collaboration for ELIXIR. The Institute was a key player in its creation and, today, SIB is ELIXIR’s largest national node.

## SIB resources, services, core facilities and training

### Resources

SIB’s groups develop, maintain and provide a large portfolio of bioinformatics resources [2] that are essential to the life science community both on the national and international level. SIB’s portal—ExPASy expasy.org—which was the first website in the biomedical field to be set up in 1993, offers access to >150 unique, high-quality databases and software tools (Table 1). The Institute’s impact is illustrated by the fact that there are few life scientists around the world who do not rely on SIB resources during the course of their research, and it is one of SIB’s missions to guarantee their long-term sustainability, for example by providing stable funding and a solid institutional environment.

**Table 1:** Examples of SIB databases and software programs for each bioinformatics domain

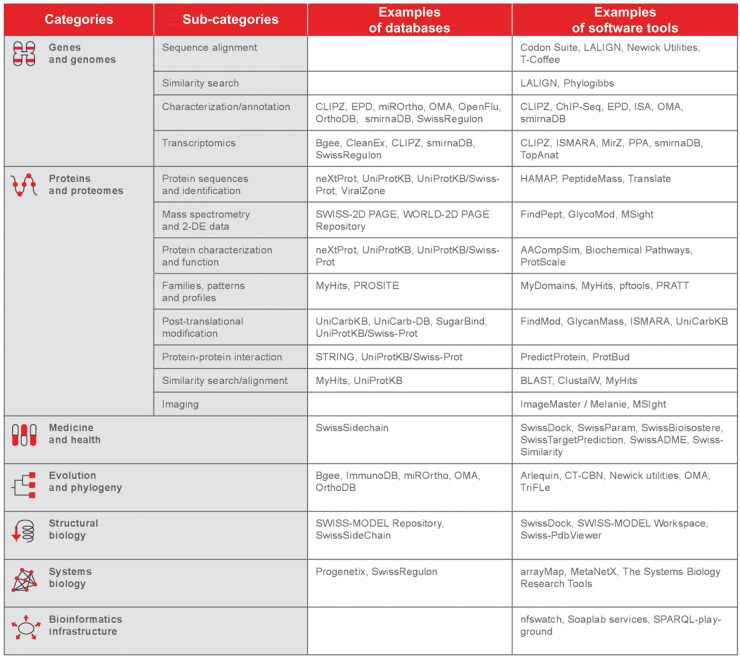

*Note*: SIB develops, supplies and maintains >150 high-quality databases and software platforms for the life science community. Most of SIB’s resources are in open access on the SIB bioinformatics resource portal ExPASy (www.expasy.org).

#### UniProtKB/Swiss-Prot

One resource in particular can be singled out for its longevity, quality, scope and impact: UniProtKB/Swiss-Prot, a resource of protein sequences and functional information. The first version of Swiss-Prot was launched in 1986, a time when few biological databases existed and when the term ‘bioinformatics’ (coined in 1970) was as yet unknown to most biologists. Swiss-Prot is the expert-curated part of the UniProt Knowledgebase (developed by the UniProt consortium, www.uniprot.org) that unites experimental results, computed features and scientific conclusions regarding proteins [3]. High-quality curated knowledge is the hallmark of Swiss-Prot, knowledge gathered by expert biocurators who make up >60% of the staff (about 40 people) of the Swiss-Prot group, working in close collaboration with curators from EMBL-EBI in the UK and Protein Information Resource in the United States. The time and labour-intensive expert curation of proteins is essential to provide the scientific community with high-quality data sets that are both reliable and programmatically parsable. UniProtKB/Swiss-Prot currently contains >550 000 manually annotated protein sequences and almost 350 000 curated literature references—data that are accessed by >900 000 requests per month on the UniProt site.

Expert curation constitutes an essential and sometimes overlooked element in the cycle of life science research and data management—including the archiving, structuring and standardizing of data and knowledge for optimal reuse. The expert-curated knowledge of Swiss-Prot is the gold standard for protein function, with a depth and accuracy which is unrivalled by any computational method (for which expert curation is both the benchmark and source of training data) [4]. Swiss-Prot also provides high coverage of the available biological knowledge, thanks to the use of effective literature triage procedures. Nevertheless, expenditure on data and knowledge curation by Swiss-Prot and other groups currently represents only a small fraction compared to total research expenditure and continues to be neglected by many funding agencies.

#### STRING-db

A second resource of importance developed under SIB leadership is STRING-db [5], a database of protein–protein interactions that are either known or that can be predicted. Several thousand organisms are represented in STRING-db, which draws its data from automated text mining, computational predictions, automated transfers of knowledge across organisms, results from high-throughput experiments and experimental interaction knowledge extracted from pathway databases. The aim of STRING-db is to cover all organisms whose genome has been fully sequenced and to report all known or predicted protein–protein interactions—in the first quarter of 2017, STRING represented 2031 organisms, 9.6 million proteins and 184 million protein–protein interactions.

STRING aggregates and integrates known and predictable protein–protein interactions, which it provides via a single, integrated user interface—something of priceless value for its users. Indeed, in this day and age of high-throughput technology, users are faced with ‘too much’ data rather than ‘too little’, and this is particularly true in the field of protein–protein interactions, which is both error-prone and context-dependent. Besides aggregating data, STRING is also a curated resource. Data curation is not carried out at the level of individual protein–protein interactions, however, but at the level of entire data collections and prediction methods in terms of how reliable they are, what weight they should get and how they can be integrated.

The resource is used by a broad audience, encompassing all life science researchers who work with molecular information. As an illustration, in 2016, the resource counted an average of 75 000 visitors and 130 000 requests on a monthly basis—with the biggest share of accesses (roughly 20% each) from the United States and China. It is also fully international, not only with regard to its users—the database is produced by a consortium of contributors from three countries: SIB in Switzerland, EMBL Heidelberg in Germany and DTU Copenhagen in Denmark.

### SIB services and core facilities

SIB provides expert data analysis services as well as a computing/storage infrastructure to life scientists in academia and industry through its bioinformatics core facilities and competence centres. Such services offer researchers the possibility to perform world-class biomedical research. To date, 12 core facilities and competence centres within the country’s borders are part of, or affiliated with, SIB.

#### Vital-IT as an example

Vital-IT is a competence centre in bioinformatics and computational biology whose users run millions of jobs every year. Its impact originates in the multidisciplinary team of skilled scientists and technical staff who enable life and medical science research in Switzerland and beyond. Its infrastructure is spread across several institutions—SIB, the Universities of Geneva, Lausanne, Fribourg and Bern, EPFL and the University hospitals of Geneva and Lausanne—each of which maintains biotechnological platforms. Vital-IT offers scientists state-of-the-art computational infrastructures—such as processing, storage and archiving—as well as expertise in data analysis and algorithmic development. It also helps scientists build computational solutions to facilitate their research or transform their ideas into production-quality software. Vital-IT’s expertise was, for example, essential in the development and improvement of the algorithm behind a novel non-invasive prenatal test (NIPT) to detect chromosomal anomalies. During the course of 2016, Vital-IT had 820 active users, and it was acknowledged in 112 publications.

#### Embedded bioinformaticians

Today, bioinformatics skills are essential in all life science projects. This is why SIB also supports Swiss universities and university hospitals by making its network and the expertise of its groups available to bioinformaticians who work within an external research or clinical laboratory. The physical presence of bioinformaticians in laboratories is an obvious advantage, as they can provide direct guidance on how to design experiments, manage and analyse data, and use various bioinformatics tools in optimal ways. In the case of personalized medicine, such a close collaboration offers an excellent opportunity to develop clinical bioinformatics tools that are especially designed for clinical research, patient data analysis, diagnosis and treatment. Currently, there are almost 30 bioinformaticians ‘embedded’ in laboratories, all of whom can benefit from SIB’s expert network.

#### SIB, a United Nations reference centre for bioinformatics

In 2015, the Food and Agriculture Organization of the UN (FAO) appointed SIB as Reference Centre for Bioinformatics—thus recognizing the Institute’s expertise and cutting-edge scientific services. In particular, SIB collaborates with FAO on the screening, monitoring and follow-up of zoonotic diseases by providing open-access databases on viruses, influenza and foot-and-mouth disease that are developed and maintained by the Swiss-Prot group and Vital-IT. As an example, in July 2012, a new avian influenza virus evolved in North America, and the sequence was published a month later. Thanks to the SIB Influenza virus database (Openflu), within an hour, it had been understood why the mutated viral gene was far more infectious than its predecessor and had caused a deadly epidemic among the local birds. FAO was able to react swiftly, thanks to the former observation of a similar mutant virus in British Columbia in 2004, which had been recorded. Their analysis was confirmed [6] months later by the scientific community.

### SIB training

The history of SIB training started with a few courses given in 1997, which became the SIB Training programme. From 1999 onwards, a full specialization in bioinformatics—an advanced MSc—was organized by SIB on behalf of the Universities of Geneva and Lausanne. As SIB grew and became central to bioinformatics in life science research nationwide, the demand for training also grew, bringing more participants and requests, and the SIB training group was finally created in December 2013 to coordinate, harmonize and expand SIB training activities. Since then, the number of courses has grown from 8 to 50 per year, reaching >1300 trainees on an annual basis. SIB training courses are tailored to attend the demand of life scientists, clinicians and bioinformaticians. They have an emphasis on practical learning, and several of them exploit SIB’s free access e-learning modules on basic bioinformatics topics. SIB training relies on an extensive combination of experts and expertise from the various SIB groups, as well as national and international speakers.

SIB training also plays an essential role in the Swiss bioinformatics training landscape, as its courses are frequently organized in collaboration with national universities and PhD programmes. Swiss universities currently provide courses in bioinformatics at the BSc and MSc levels as well as PhD programmes in the life sciences. SIB complements this offer with courses for PhDs, postdocs and researchers, thus ensuring the best use of bioinformatics and SIB’s resources. The Institute also runs the SIB PhD Training Network, the single exclusively bioinformatics-oriented doctoral programme in Switzerland, which specifically targets Swiss bioinformatics and computational biology PhD students.

Today, SIB’s training portfolio has an extensive number of bioinformatics and data science courses, involving computational biology methods, statistics, machine learning, computing techniques, as well as the analysis, management and reproducibility of biological data applied to the life sciences.

## SIB today: a few examples of applied bioinformatics

### 

Currently, SIB offers the life science community bioinformatics resources, which cover a wide array of activity domains in the fields of genomics, proteomics, medicine and health, evolution and phylogeny, structural biology and systems biology—besides providing one of the most important and long-standing bioinformatics infrastructures in the country [2]. Thanks to such resources, major advances have been made. Among them, three are of note: prenatal diagnostics, 3D visualization and clinical bioinformatics.

#### Prenatal diagnostics: a revolutionary test

The world of NIPTs—i.e. tests that are not carried out on the placenta, for instance, as is the case in amniocentesis—is revolutionizing prenatal diagnostics. Compared with traditional amniocentesis tests, which can only be carried out from the 15th week of pregnancy, results for non-invasive tests can be executed as early as the 9th to the 10th week of pregnancy and, unlike amniocentesis, there is no risk of miscarriage. What is more, results come in faster with an average of 5 days for non-invasive tests against 2 weeks for amniocentesis tests. This is because prenatal non-invasive tests are carried out on foetal cell-free DNA, which is found in trace amounts in the bloodstream of pregnant women [7]. Foetal DNA levels as low as 5% with respect to maternal DNA permit the reliable detection of common aneuploidies using high-throughput sequencing.

SIB developed an algorithm behind an NIPT able to detect chromosomal anomalies—such as trisomy 21 for instance—in foetal cell-free DNA [8]. Trisomy 21 is a well-known chromosomal anomaly; there are other less frequent trisomies, however, such as trisomies 18 and 13 that involve severe intellectual disability as well as health problems for nearly every organ, and most babies die within the first year of their life. It turned out that despite the fact that chromosome 21 trisomy is relatively easy to detect, the other trisomies were more difficult to identify and required the development of highly specific analysis techniques.

Although NIPT tests were initially targeted to give solid information on these three recurrent chromosomal anomalies, SIB researchers were able to fine-tune their algorithms further to detect all aneuploidies and sub-chromosomic anomalies, such as local duplications or deletions, that are rare and equally severely disabling. They also introduced into the algorithm a direct estimation of the cell-free foetal DNA present in the sequencing data used to detect anomalies—a far more robust way of assessing the quality of the test than using a secondary assay [7].

#### A 3D visualization of molecules

Molecular modelling, in general, and computer-aided molecular design, in particular, are important fields of activity within SIB, and many of its molecular design software tools such as SWISS-MODEL [9], Swiss-PdbViewer [10] and SwissDock [11] are used by scientists on a daily basis around the globe. All three tools have been cited >10 000 times in scientific publications over the past 20 years, with an average of about 550 citations a year, according to Thomson Reuters. Molecular modelling enables researchers not only to visualize biological molecules of any nature in 3D format on a screen but also to modify them virtually and see what such modifications could imply. As an example, scientists can visualize 3D models of protein structures and simulate their function in their natural state, but also find out what happens to them when a mutation occurs and is harmful to an organism, as is the case in cancer. With such knowledge, scientists can then design anti-cancer drugs *in silico* and study the interactions that are expected to occur to counter the harmful effects of a mutation.

SIB is renowned for powerful drug design software to guide the design of new small bioactive compounds. These tools cover not only structure-based drug design, which make use of the 3D structure of the targeted protein-like docking programmes, but also ligand-based approaches that extract information from known ligands and substrates of the protein using machine learning algorithms. The collection of SIB structure-based drug design tools contains SwissDock, a free, simple and professional docking Web service based on our EADock DSS [12] docking engine; SwissParam [13] that provides topologies and parameters for the molecular modelling of small organic molecules; and finally SwissSidechain [14], a database that centralizes information on hundreds of commercially available non-natural amino acids for peptide design [15]. SIB ligand-based drug design tools regroup SwissBioisostere [16], a comprehensive and freely accessible database of 4.5 million molecular replacements experimented in small molecule optimization; SwissTargetPrediction to predict the targets of bioactive small molecules in human and other vertebrates for toxicity prediction or drug repurposing; SwissADME to estimate physico-chemical properties of organic molecules in relation with their pharmoco-kinetic, pharmaco-dynamic and druglikeness properties, and finally SwissSimilarity that allows the rapid virtual screening of large-scale libraries of drugs, bioactive small molecules, commercially available compounds and an unprecedented large number of virtual compounds readily synthesizable from commercially obtainable reagents. These services are fully automated and combine accurate algorithms with interfaces that are especially designed for users who are not bioinformaticians. Such software does not replace *in vitro* or *in vivo* tests, but it does provide rational guidance and diminishes unnecessary tests, and should decrease the time required to design a drug and its overall research and development cost that is estimated to lie between 400 and 2600 million dollars—75% of which [17] is explained by failures during the drug discovery and development process.

One interesting application is in the field of immuno-oncology. Indoleamine 2,3-dioxygenase, or IDO1, is an enzyme known to play a major role in tumour-induced immunosuppression. One of the computational drug-design tools developed within SIB allowed the design of highly efficient IDO1 inhibitors both in enzymatic and cellular assays as well as *in vivo*, while showing no cellular toxicity and a high selectivity for IDO1 over related enzymes [18]. Such studies should ultimately lead to substantial and long-term clinical benefits for cancer patients.

### Clinical bioinformatics

Besides data pertaining to a patient’s lifestyle, eating habits and vital signs, medical practitioners are now increasingly faced with complex molecular data such as their patients’ genomic sequence, proteomic profile or metabolic profile. The analysis, integration and interpretation of these complex data require a deep understanding of their biological meaning, coupled to a specific expertise on algorithms and data analysis methods. This is where bioinformatics plays a key role in supporting clinicians in their daily decisions by providing the right set of expertise to help them to interpret such complex data. In the not too distant future, a clinician will be able to use these data to diagnose a disease and suggest a treatment that is not only suited to a given disease but even tailored to the specific patient—this is the realm of personalized health. For this to happen in the most effective manner, however, bioinformatics tools need to be easy to use and adapted to medical practice, taking into account clinical-specific needs and constraints.

In 2012, SIB created a Clinical Bioinformatics Group to provide clinicians with bioinformatics expertise to organize, analyse and interpret patient-related genomics, proteomics and metabolomics data. The aim is to harmonize, standardize and coordinate the bioinformatic analysis of high-throughput data in hospitals across the country. Country-wide working groups in Somatic mutation calling and Microbe typing and characterization have been set up by SIB to establish a collaborative framework between clinicians and bioinformaticians. This contributed to identifying common best practices and defining clinically useful bioinformatics tools. A detailed survey of current next-generation sequencing (NGS) practices was also sent to those involved in the Somatic mutation calling working group. The information gathered led to a round robin trial that aims to identify common best practices among Swiss hospitals, and optimize existing pipelines. A detailed questionnaire on NGS practices for bacterial typing and viral metagenomics has also been submitted to the Microbe typing and characterization working group to assess current clinical practices on the national level.

By the end of 2015, the collaboration between the Geneva University Hospitals (HUG) and SIB was formalized, and in 2016, OncoBench^TM^ was launched: a bioinformatics analysis tool for the routine molecular diagnosis of cancer patients, based on NGS data. OncoBench^TM^ is an optimized Web-based application that streamlines the identification of somatic variants, and stores all the related information in a structured way using ontologies and controlled vocabularies, thus ensuring data standardization and traceability. Subsequent to a satisfaction survey conducted on the end users of the HUG Clinical Pathology Service, the application notably allows for a gain in time (turnaround, from sample to report, of 4 days instead of 5), in addition to an estimated timesaving of 1 full day per week for the laboratory. The application is running on a local high-performance computing infrastructure, where all data are stored and processed. This computing cluster is managed by SIB, in collaboration with the hospital IT services, and is embedded on the hospital’s premises, therefore ensuring complete patient data privacy—a matter of significant importance. What is more, being structured and standardized, the resulting data repository also facilitates future data searching and sharing. It is therefore significant within the implementation of the SPHN whose ambition is to make patient data across the country securely available to research projects, and in which SIB plays a pivotal role.

## SIB: structure and organization

SIB has become a reference throughout Europe with regard to its structure and organization, and the Institute is frequently approached by countries seeking guidance to create their own national bioinformatics infrastructure. Questions cover operational, technical, governance and strategic perspectives. How does SIB operate given its decentralized character? What are the difficulties encountered with regard to synergies in investments? What is required to become an SIB member? What is the hardware architecture of SIB? How are SIB services accessed? These are just a few of the questions, many of which have already been addressed here. It is not possible to deal with them in detail, but there are a few things that are either unique or essential to an organization such as SIB.

### SIB organization

From an organizational perspective, as a non-profit foundation, the Institute has a Foundation Council (FC) composed of representatives of all partner institutions, which names its Group Leaders (GLs), the Board of Directors (BoD), and the Scientific Advisory Board (SAB). The FC adopts the yearly budget and financial report, and agrees on the general strategy to follow. The BoD defines the general strategy, ensures that the goals of the Institute are met and is responsible for allocating federal funds based on SAB recommendation.

The management team has a current staff of almost 20 collaborators. SIB has an executive office, a finance and grant department, a legal and technology transfer officer, a human resources department and a communications department. In addition to the SIB-wide activities in Training and Clinical Bioinformatics, the Institute has a Technology group that coordinates and optimizes technical activities across SIB. SIB members can refer to any of these instances for help or information, and ask the communications department for assistance in writing up press releases or any other document relative to broadcasting their work.

### Requirements to become a GL at SIB

A number of criteria need to be met to become a GL at SIB. In particular, an aspiring GL must have a professorial appointment in a partner institution or be a senior scientist who leads their own group. As SIB does not finance GLs, they must have a sufficient level of financial independence, such as managing their own budget and/or holding research grants. The group’s activities must also lie within SIB’s definition of bioinformatics in terms of people, publications and/or funding. Furthermore, to date, only groups who are actively involved in developing bioinformatics methods and software or managing core facilities are taken into consideration, as opposed to groups who ‘simply’ make extensive use of *in silico* approaches, for example. If a group runs a core facility that provides databases or bioinformatics support, the mission and activities of the group as a whole are evaluated, rather than the research credentials of the GL. The group cannot be exclusively a Wet laboratory that has some embedded bioinformatics activities; if this is the case, the embedded bioinformaticians can affiliate to an existing SIB group. A number of secondary self-evident criteria are also required. The activities carried out by a GL have to be of interest to the Institute, and their academic credentials of international status. All GLs are expected to take part in SIB’s activities, as well as support and defend the Institute’s mission.

### SIB bioinformatics resources: selection process

The long-term sustainability of SIB’s core resources is one of SIB’s *raison d’être*. Each of the Institute’s resources is evaluated according to objective indicators that have been established and refined over a period of 20 years, and are followed rigorously. These indicators evaluate essential aspects such as scientific excellence (data quality; uniqueness and comprehensiveness; benchmarking information), the community served (usage numbers; citations; national and international usage; dependencies of other resources), user feedback, the resource’s impact (the resource’s importance for the life science community; strategic importance of the resource for SIB; and how the community or SIB would be impacted if it were to disappear).

An external SAB advises SIB’s BoD on which resources should be maintained and what the Institute’s strategy should be. Furthermore, even though SIB can take the decision to support a new resource, it can also decide to discontinue or diminish any support for an existing Core Resource if its impact has significantly weakened. To foster innovation, SIB also has a limited budget that is distributed in a competitive manner to emerging resources.

### SIB finance

The total sum of funds managed by SIB in 2016 amounted to CHF 27.3 million. The largest source is the Swiss government, which provides 42.1% of SIB’s total income. Other funds come from the Swiss National Science Foundation and European funds (11.7%), the National Institutes of Health (9.5%), SystemsX.ch (4.4%), the industry (11.0%), Universities and hospitals (16.5%) and a few others. In all, in 2016, 51 competitive grants/contracts were managed by SIB. The total 2016 budget for bioinformatics in Switzerland (including funds managed by SIB and funds managed by SIB’s partner institutions) amounted to CHF 82.7 million. Of the total amount spent by SIB in 2016, 83% was allocated to salaries, and the rest to equipment (4.6%), scientific events (1.1%) and running costs (11.3%).

### Member benefits

Becoming an SIB member offers many benefits: researchers are part of a leading national bioinformatics network, thus easing the flow of information between the groups and offering opportunities to interact by creating special interest groups, for instance. One illustration is PhyloSIB, an annual workshop that has met with much success and gathers SIB groups with an interest in molecular evolution, phylogenetics and comparative phylogenomics to exchange knowledge and discuss ongoing studies and developments in phylogeny.

Besides workshops and special groups, all SIB members are invited to the ‘SIB Days’, a biannual opportunity to discuss work, promote collaborations and foster a sense of community. The programme always features eminent keynote speakers, presentations of the latest scientific results of SIB research groups as well as workshops and poster sessions.

### Essential ingredients

If there are points to stress for countries that are thinking of creating an institute based on a structure such as SIB’s, they would be to focus on long-term sustainability; form an SAB, which is external; and recognize the importance of training programmes.

## Looking ahead

SIB has pioneered bioinformatics in Switzerland by leading the field and providing exceptional resources to the life science community both on the national and international level. It was founded at the best of times, when bioinformatics had become a scientific discipline *per se*, but the field had not yet revolutionized the life sciences as a whole. SIB grew fast. Its structure is unique: though decentralized, it federates bioinformatics throughout the country while promoting collaboration between researchers, and it has had plenty of time to mature.

Today, the Institute federates many important research and service groups, creating a dynamic bioinformatics network across the whole country. It has become a role model and inspired the structure of organizations outside the country’s borders such as ELIXIR. The European bioinformatics landscape is changing, and instances like ELIXIR are inspiring other countries to create their own institute based on SIB’s organizational model.

On the medical front, SIB acknowledges the fact that the wealth of data produced by modern technologies in medicine and the growing self-awareness of patients is about to revolutionize the way of considering medical data. The Institute will pursue its efforts in clinical bioinformatics embracing the challenge of excellence in bioinformatics for personalized health, thus working towards a more global approach of patients, and more targeted and effective therapies. SIB will continue its major role in the SPHN to make patient-related data available to research across the country.

The SIB adventure began when one core resource was at stake. Yet, at the time, the resource was used worldwide by the life science community and continues to be so 20 years later—this illustrates how important it is to secure funds on a long-term basis. It is one of SIB’s daily challenges: to maintain its current data resources and software tools while fostering innovation and novel high-quality resources on a limited budget. It is also important to know how to identify and finance the most crucial resources, whose impact is beneficial in as many ways possible. The long-term sustainability of its resources is one of SIB’s priorities, and must remain so for the benefits of research in all fields of the life sciences. It is equally important to keep demonstrating the worthiness of being part of an institute such as SIB, for the advancement of bioinformatics on the national and the international level—and not only to those who are already members but also to research groups who are perhaps considering membership. A federation of research and service groups such as SIB is ideal for creating collaborations and interactions with an ever-growing number and diversity of groups and members nationwide and, though the task is not always an easy one, the Institute continuously encourages its members in this respect. Hence, 2018 marks the Institute’s 20th anniversary—with time and determination, the Institute has acquired a leading position in supporting and offering assistance in bioinformatics across Switzerland and beyond. In the years to come, SIB will continue to consolidate its pivotal role in providing a bioinformatics environment for the advancement of research in the life and medical sciences.

##

**Key Points**

- The SIB federates some 65 research groups, service groups and competence centres across the country.
- SIB makes available over 150 high-quality databases and software tools used by life scientists worldwide.
- SIB provides expert data analysis services and computing/storage infrastructure to life scientists in academia and industry through its bioinformatics core facilities.
- SIB offers training courses tailored to the needs of life scientists, clinicians and bioinformaticians.
- SIB has a leading role in the Data Coordination Centre for the SPHN—a national infrastructure for the sharing of patient data for research across Switzerland.
